# Identification of Hub Genes in Idiopathic Pulmonary Fibrosis and NSCLC Progression:Evidence From Bioinformatics Analysis

**DOI:** 10.3389/fgene.2022.855789

**Published:** 2022-04-11

**Authors:** Yuanshan Yao, Zheng Li, Wen Gao

**Affiliations:** Department of Thoracic Surgery, Shanghai Key Laboratory of Clinical Geriatric Medicine, HuaDong Hospital Affiliated to Fudan University, Shanghai, China

**Keywords:** hub genes, idiopathic pulmonary fibrosis, lung cancer progression, bioinformatics analysis, pathways

## Abstract

**Background:** Lung cancer is the most common comorbidity of idiopathic pulmonary fibrosis. Thus there is an urgent need for the research of IPF and carcinogenesis

**Objective:** The objective of this study was to explore hub genes which are common in pulmonary fibrosis and lung cancer progression through bioinformatic analysis.

**Methods:** All the analysis was performed in R software. Differentially expressed genes (DEGs) were explored by comparing gene expression profiles between IPF tissues and healthy lung tissues from GSE24206, GSE53845, GSE101286 and GSE110147 datasets. Venn Diagram analysis was used to identify the overlapping genes, while GO and KEGG pathway enrichment analysis were used to explore the biological functions of the DEGs using clusterprofiler package. Hub genes were identified by analyzing protein-protein interaction networks using Cytoscape software. Nomogram was constructed using the rms package. Tumor immune dysfunction and exclusion (TIDE) and Genomics of Drug Sensitivity in Cancer (GDSC) analysis was used to quantify the immunotherapy and chemotherapy sensitivity of non-small cell lung cancer (NSCLC) patients.

**Results:** *COL1A1, COL3A1, MMP1, POSTN1* and *TIMP3* were identified as the top five hub genes. The five hub genes were used to construct a diagnostic nomogram that was validated in another IPF dataset. Since the hub genes were also associated with lung cancer progression, we found that the nomogram also had diagnostic value in NSCLC patients. These five genes achieved a statistically difference of overall survival in NSCLC patients (*p* < 0.05). The expression of the five hub genes was mostly enriched in fibroblasts. Fibroblasts and the hub genes also showed significant ability to predict the susceptibility of NSCLC patients to chemotherapy and immunotherapy.

**Conclusion:** We identified five hub genes as potential biomarkers of IPF and NSCLC progression. This finding may give insight into the underlying molecular mechanisms of IPF and lung cancer progression and provides potential targets for developing new therapeutic agents for IPF patients.

## Background

Idiopathic pulmonary fibrosis (IPF) is a fatal interstitial pneumonia mostly occurring in old patients. It is characterized by progressive, chronic, and irreversible diffuse pulmonary fibrosis. Pathological results show diffuse alveolitis and disordered alveolar structural, which eventually lead to the destruction of alveolar structure to form honeycomb lung (Wolters et al., 2014). Patients often complain of progressive breathlessness and consistent cough for years, with respiratory failure appearing at the end stage (Lederer and Martinez, 2018). High resolution CT of chest is necessary for the diagnosis of IPF, and a thoracoscopic lung biopsy is recommended to obtain some lung tissues for further individualized therapy. Although the exact pathogenesis of IPF is not completely understood, it is generally acknowledged that genetic susceptibility and some factors such as smoking, drug, and viral exposures lead to alveolar remodeling and regulate fibroblasts clustering, ultimately forming progressive fibrosis (Somogyi et al., 2019). Previous studies showed that lung cancer is the most common complications of IPF, with IPF increasing the risk of developing lung cancer by 20% (Ballester et al., 2019). Lung cancer in IPF patients is associated with poor prognosis, and greater incidence of squamous lung cancer, unlike lung cancer in patients without IPF (Tzouvelekis et al., 2018).

Several lung cancer associated genes, such as *TP53*and *KRAS*, are closely related with IPF (Tzouvelekis et al., 2019). *miR-21* has been displayed to promote lung cancer progression and its expression levels are up regulated in IPF and lung cancer (Liu et al., 2010; Bica-Pop et al., 2018). Hence, there is need to develop novel therapeutic agents to treat patients with IPF and coexisting lung cancer. Anti-fibrosis drugs including Pirfenidone and nintedanib have been approved for the treatment of IPF and can slow the disease progression, but their efficacy is limited and cannot reverse the effects of the disease (Richeldi et al., 2014; Kang and Song, 2021). There is an increase in cases of end-stage IPF patients undergoing lung transplantation due to poor lung function. Therefore, it is possible for doctors to obtain whole IPF samples and identify the gene expression profile through high throughput sequencing. Bioinformatic tools can facilitate the discovery of new hub genes closely associated with IPF and lung cancer progression.

In our study, we applied bioinformatic algorithms to explore the common hub genes and biological pathways associated with IPF and lung cancer progression. The findings from this study may increase the understanding of the underlying molecular mechanisms of IPF and lung cancer and identify new targets for the development of new therapeutic agents for patients with IPF and coexisting lung cancer.

## Materials and Methods

### RNA Expression Datasets

The raw data used in this study were retrieved from gene expression omnibus (GEO) and The cancer genome atlas (TCGA). IPF gene expression datasets GSE24206 (Platform: GPL570, Affymetrix Human Genome U133 Plus 2.0 Array), GSE53845 (Platform: GPL6480, Agilent-014850 Whole Human Genome Microarray 4 × 44K G4112F), GSE101286 (Platform: GPL6947, Illumina HumanHT-12 V3.0 expression beadchip) and GSE110147 (Platform: GPL6422, Affymetrix Human Gene 1.0 ST Array) consisting of 17 IPF samples, 40 IPF samples, 12 IPF samples, and 22 IPF samples, were downloaded from GEO, respectively. Only the data of the IPF samples and normal healthy lung tissue were included in our analysis. Lung cancer gene expression datasets were downloaded from TCGA. Data for 513 adenocarcinoma and 501 squamous carcinoma patients who had information on survival time and survival status were included in this study (TCGA-LUAD and TCGA-LUSC, Data Category: transcriptome profiling; workflow type: HTSeq-FPKM). The clinical information was downloaded with the “bcr xml” form and collated using Perl code. The GSE32539 dataset was also downloaded and used as a validation set to verify the efficacy of the diagnostic model based on the hub genes, containing 119 IPF patients.

### Data Processing and Identification of Differentially Expressed Genes

Background correction and normalization were performed before the DEGs analysis using limma package and the codes was uploaded in figshare (https://figshare.com/articles/dataset/Original_document/19299518) (Ritchie et al., 2015). Then, limma package was used to identify DEGs between IPF and normal tissues in GSE24206, GSE53845, GSE101286 and GSE110147 datasets. DEGs were defined using the following criteria: *p* < 0.05 and |logFC|>1 (Ritchie et al., 2015). Then Venn analysis was performed to explore overlapped DEGs among the four datasets (http://bioinformatic.psb.ugent.be/webtools).

### PPI Network Establishment and Hub Gene Exploration

Protein-protein interaction (PPI) network for the previously identified DEGs was built using The Search Tool for the Retrieval of Interacting Genes (STRING) and visualized through cytoscape software (www.cytoscape.org/). The hub genes were identified through a novel plugin in Cytoscape v3.7.2 software, named CytoHubba. CytoHubba plugin is implemented in Java (Cytoscape API) and quantify the importance of nodes in biological interaction network through 11 node ranking methods, including Degree, Edge Percolated Component, Maximum Neighborhood Component, Density of Maximum Neighborhood Component, Maximal Clique Centrality (proposed in this paper), Bottleneck, EcCentricity, Closeness, Radiality, Betweenness, and Stress (Chin et al., 2014).

### Biological Enrichment Pathways

GO and KEGG enrichment analyses were used to analyze the biological pathways of these core genes through the Clusterprofiler package in R environment (Yu et al., 2012). Enriched GO terms were stratified into cell components (CC), biological process (BP) and molecular function (MF) terms. The organism reference was set as “org.Hs.eg.db”. The terms meeting the cutoff of pvalue <0.05 and qvalue <0.05 were selected.

### Analysis of Single Cell Data

The human cell landscape analysis tool (bis.zju.edu.cn/HCL) was used to explore the expression landscape of five hub genes in three human normal lung tissues from previously published single cell datasets (Strack, 2020).

### Clinical Correlation

Survival analysis was carried out using Kaplan-Meier method based on the five hub genes. Bioinformatic analysis was used to identify any associations between the hub genes and clinicopathological characteristics of NSCLC patients. The patients were stratified based on age, gender, TNM stage, T stage, N stage, M stage, and smoking history. The data was first stratified into two groups based on the levels of cancer-associated fibroblasts. Kaplan–Meier curves were then used to compare overall survival and disease free survival between the two groups through Gene Expression Profiling Interactive Analysis (GEPIA http://gepia.cancer-pku.cn/) (Tang et al., 2017). Statistically significant differences between curves were determined using the log-rank test.

### Establishment and Validation of a Diagnostic Nomogram for IPF and NSCLC

A diagnostic nomogram was established based on the hub genes by using rms package in R software. The receiver operating characteristic curve was used to investigate the efficiency of this diagnostic model. Area under curve >0.7 was considered significant.

### Immunotherapy and Chemotherapy Sensitivity

To establish the functions of the five hub genes, we explored the association between the genes and the sensitivity of immunotherapy and chemotherapy. Possible response to immunotherapy was evaluated through Tumor Immune Dysfunction and Exclusion (TIDE) algorithm (tide.dfci.harvard.edu) (Jiang et al., 2018), while potential response to cisplatin- and paclitaxel-based chemotherapy was estimated using genomics of drug sensitivity in cancer (GDSC) algorithm (http://www.cancerrxgene.org) (Yang et al., 2013).

### Statistical Analysis

Statistical analyses were carried out in R software v4.0.5. *p* value was two-sided and less than 0.05 was considered statistically significant.

## Results

### Identification of DEGs

**FIGURE 1:**
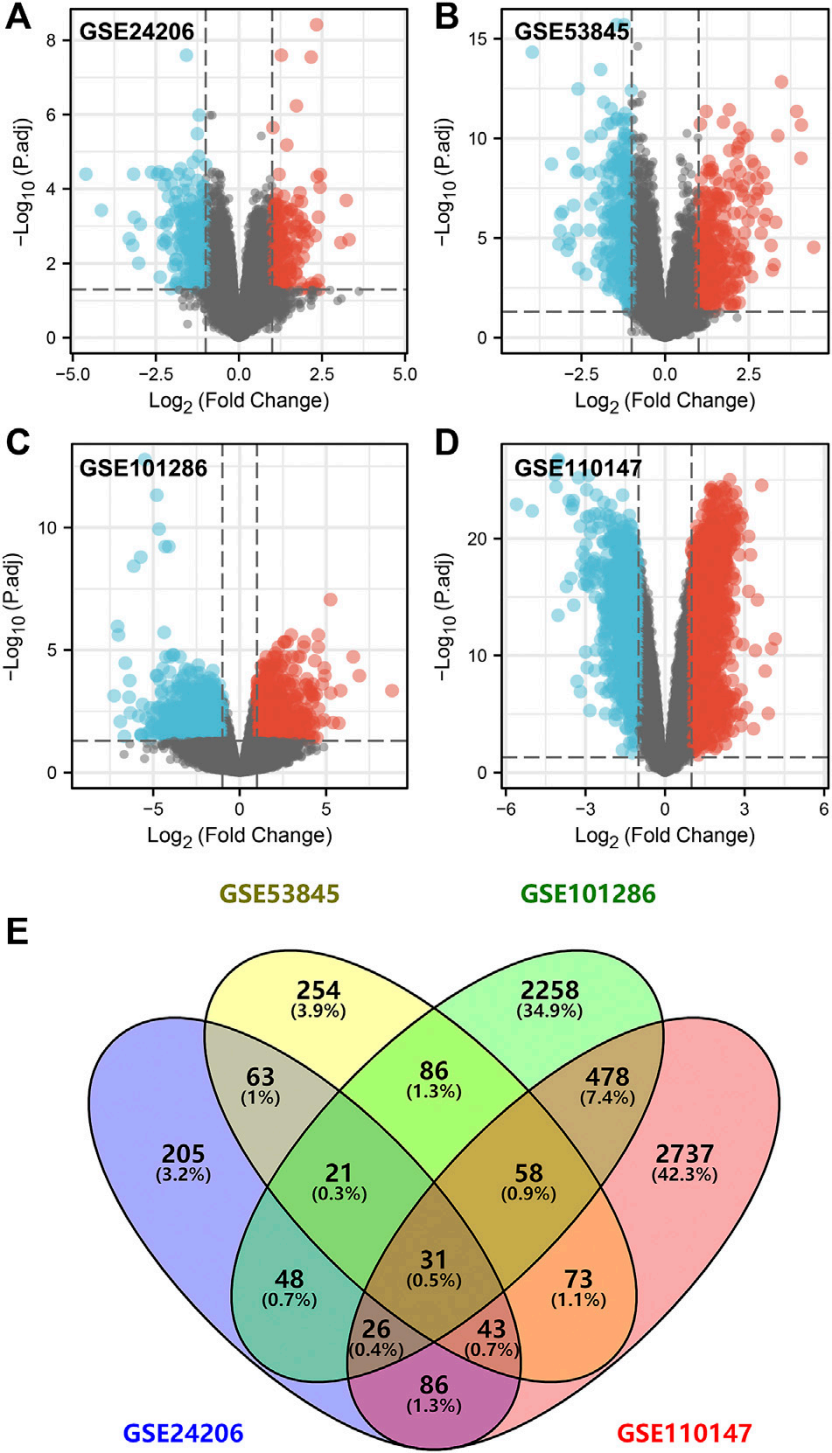
Identification of DEGs in IPF patients. Notes: **(A–D)**: DEGs analysis was performed between IPF and normal lung tissue with the threshold of *p* < 0.05 and |logFC| > 1 based on the GSE24206, GSE53845, GSE101286 and GSE110147; **(E)**: A Venn graph analysis intersected 31 common DEGs in GSE24206, GSE53845, GSE101286 and GSE110147 database.

Table 1 comprehensively summarized the results. Detailed, four raw IPF gene expression datasets (GSE24206, GSE53845, GSE101286, GSE110147) were included in our study. After background correction and data standardization (Figure S1), DEGs between IPF and normal lung tissues were identified using a criteria of adjusted *p* < 0.05 and |logFC|>1.0. Volcano plots were established using fold-change values and adjusted P (Figures 1A–D). The number of DEGs identified were 523 (271 up regulated and 252 down regulated genes), 629 (356 up regulated and 273 down regulated genes), 3,006 (1,457 up regulated and 1,549 down regulated) and 3,532 (2,239 up regulated and 1,293 down regulated genes) from GSE24206, GSE53845, GSE101286, and GSE110147 datasets, respectively (Figures 1A–D). Venn analysis identified 31 DEGs whose expression overlapped among the four datasets (Figure 1E and Table S1).

**TABLE 1:** The overview of the dataset used for this study.

Dataset	Platform	IPF Patients	Normal Patients	Used for	Upregulated Genes (under the Threshold)	Downregulated Genes (under the Threshold)
GSE24206	GPL570	17	6	DEGs analysis	271	252
GSE53845	GPL6480	40	8	DEGs analysis	356	273
GSE101286	GPL6947	12	3	DEGs analysis	1,457	1,549
GSE110147	GPL6422	22	11	DEGs analysis	2,239	1,293
GSE32539	GPL6244 (mRNA part)	119	50	Model validation	Not applicable	Not applicable

### PPI Network Construction and Hub Gene Identification

**FIGURE 2:**
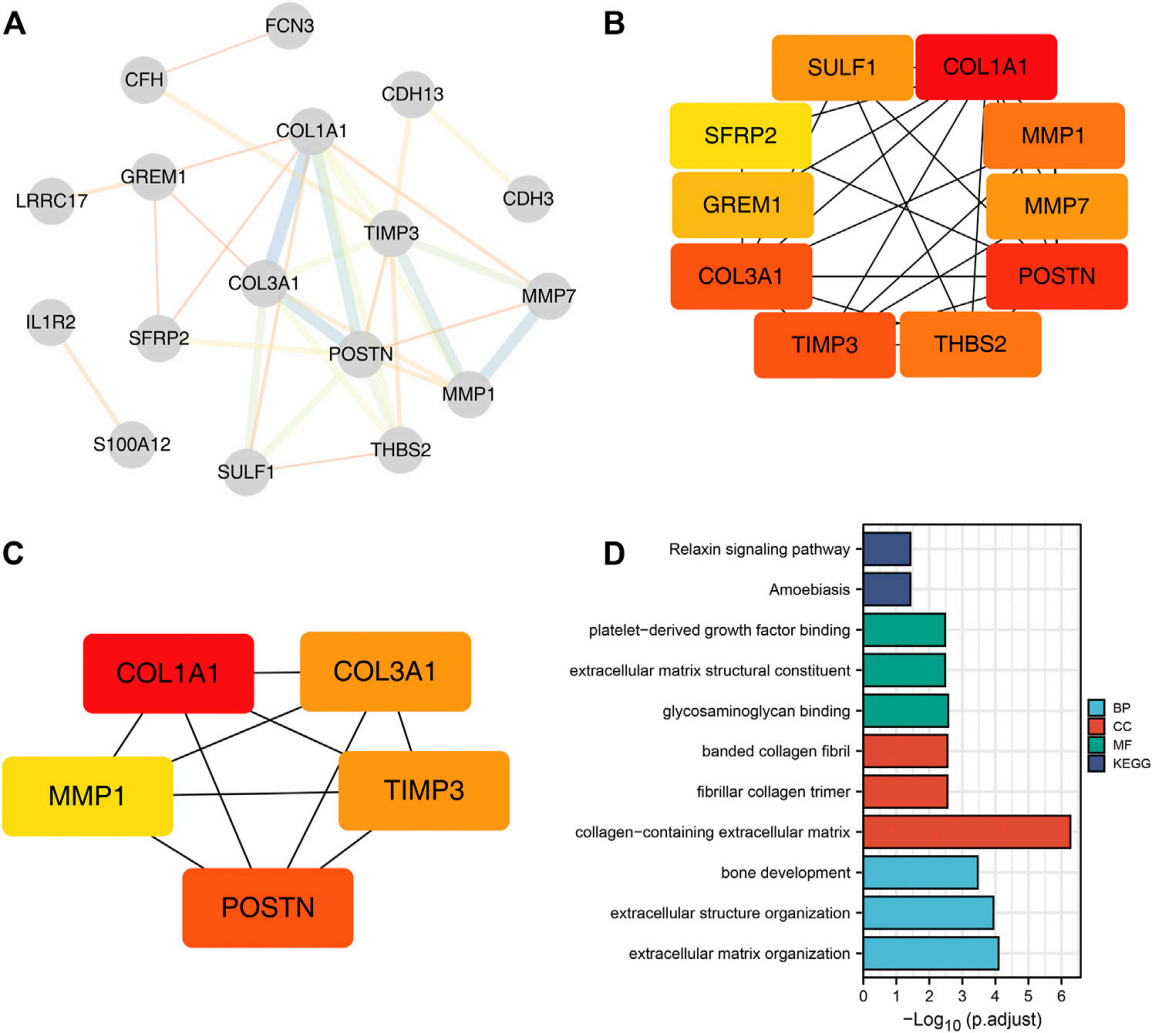
Five hub genes were identified in IPF samples. Notes: **(A)**: PPI network of the DEGs; **(B)**: Top ten central elements calculated by connectivity degree in the PPI network; **(C)**: Top five central elements named hub genes calculated by connectivity degree in the PPI network; **(D)**: Gene Ontology (GO) and Kyoto Encyclopedia of Genes and Genomes (KEGG) enrichment pathways were explored based on the 31 common DEGs.

Using STRING tools, the PPI network was found to have 31 nodes and 33 edges (Figure 2A). The top ten central elements calculated by degree of connectivity in the PPI network are shown in Figure 2B, while the top five central genes in the PPI network termed the hub genes are shown in Figure 2C. The five hub genes in IPF were: *COL1A1, COL3A1, MMP1, POSTN1*, and *TIMP3*.

### Biological Enrichment Pathways

GO and KEGG enrichment pathways were explored through the Clusterprofiler package by using the above 31 DEGs (Figure 2D and Supplementary Table S2). Enriched BP pathways included extracellular matrix organization, extracellular structure organization and bone development. Results of CC analysis showed collagen-containing extracellular matrix, fibrillar collagen trimer and banded collagen were the most enriched cell components. MF analysis revealed that glycosaminoglycan binding, extracellular matrix structural constituent and platelet-derived growth factor binding were the most enriched biological pathways. Moreover, KEGG results indicated that DEGs were closely associated with amoebiasis and relaxin signaling pathways.

### Hub Gene Expressions Analysis in IPF and NSCLC

**FIGURE 3:**
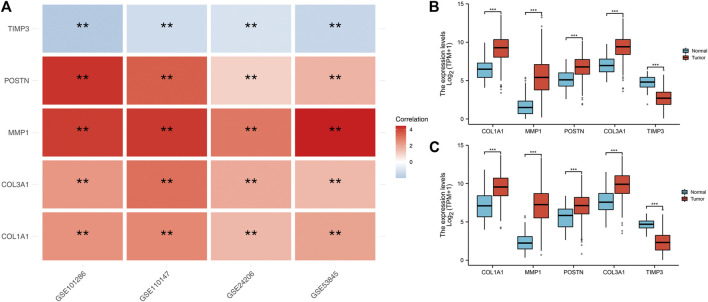
Expression level of the five hub genes in IPF and NSCLC patients. Notes: **(A)**: Heatmap comparing hub gene expression levels between IPF tissue and normal lung tissue in GSE24206, GSE53845, GSE101286 and GSE110147 datasets, ns = *p* > 0.05, * = *p* < 0.05, ** = *p* < 0.01, *** = *p* < 0.001.; **(B)**: The expression difference of hub genes between cancer tissue and normal lung tissue in TCGA-LUAD patients, ns = *p* > 0.05, * = *p* < 0.05, ** = *p* < 0.01, *** = *p* < 0.001.; **(C)**: The expression difference of hub genes between cancer tissue and normal lung tissue in TCGA-LUSC patients, ns = *p* > 0.05, * = *p* < 0.05, ** = *p* < 0.01, *** = *p* < 0.001.

We found that *COL1A1, COL3A1, MMP1* and *POSTN1* expression levels were significantly up regulated, while *TIMP3* was significantly down regulated in IPF patients (*p* < 0.01). We then compared the expression levels of the hub genes between TCGA-LUAD and TCGA-LUSC tissues and normal tissues. Interestingly, the expression levels of the hub genes showed a similar trend in NSCLC tissue as in IPF tissues (Figures 3B,C).

Analysis of the association between the five hub genes and survival and clinicopathological features of NSCLC patients.

**FIGURE 4:**
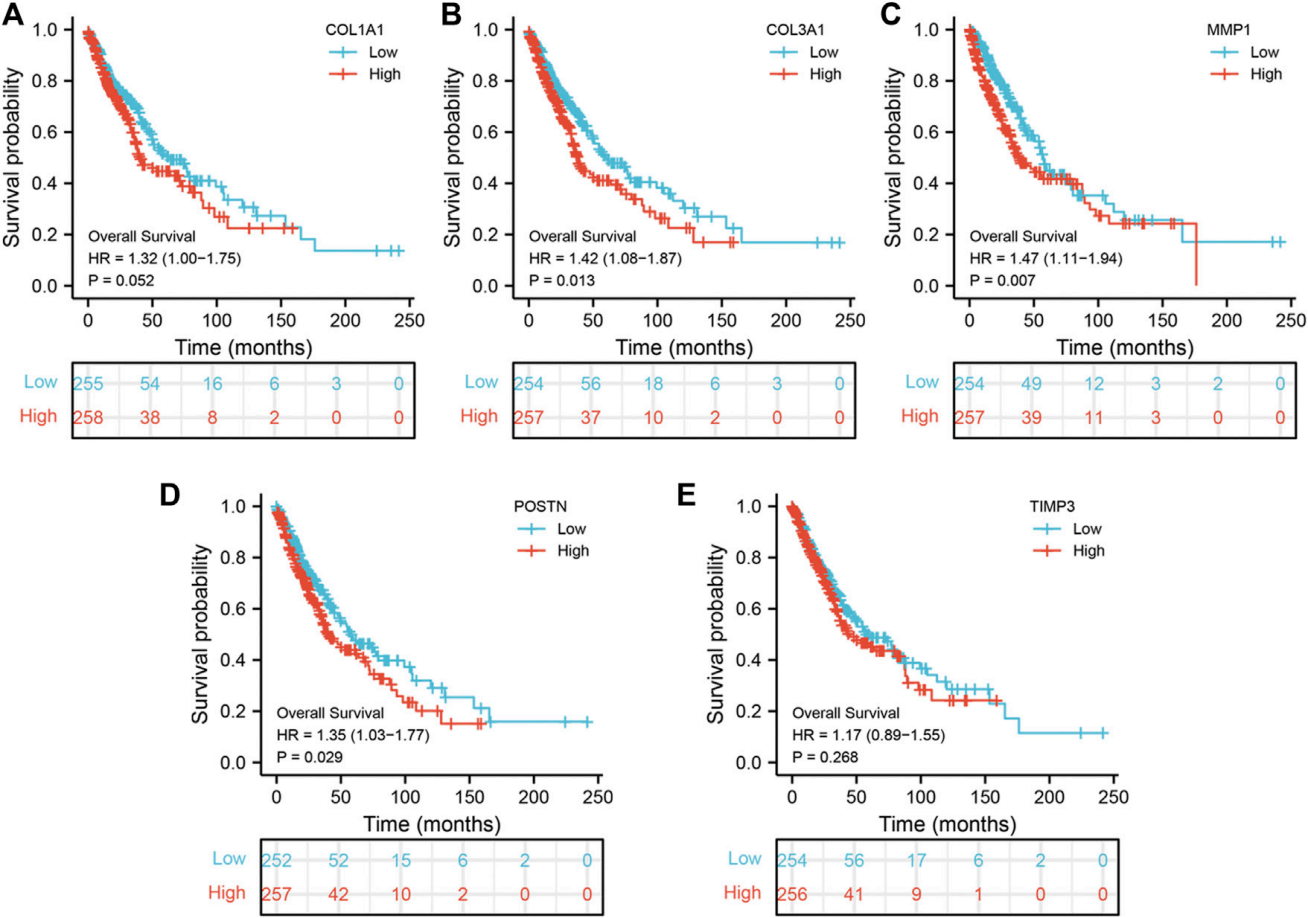
Prognosis value of hub genes. Notes: **(A)**: Kaplan-Meier curves showed the prognosis value of *COL1A1* in NSCLC patients; **(B)**: Kaplan-Meier curves showed the prognosis value of *COL3A1* in NSCLC patients; **(C)**: Kaplan-Meier curves showed the prognosis value of *MMP1* in NSCLC patients; **(D)**: Kaplan-Meier curves showed the prognosis value of *POSTN* in NSCLC patients; **(E)**: Kaplan-Meier curves showed the prognosis value of *TIMP3* in NSCLC patients.

**FIGURE 5:**
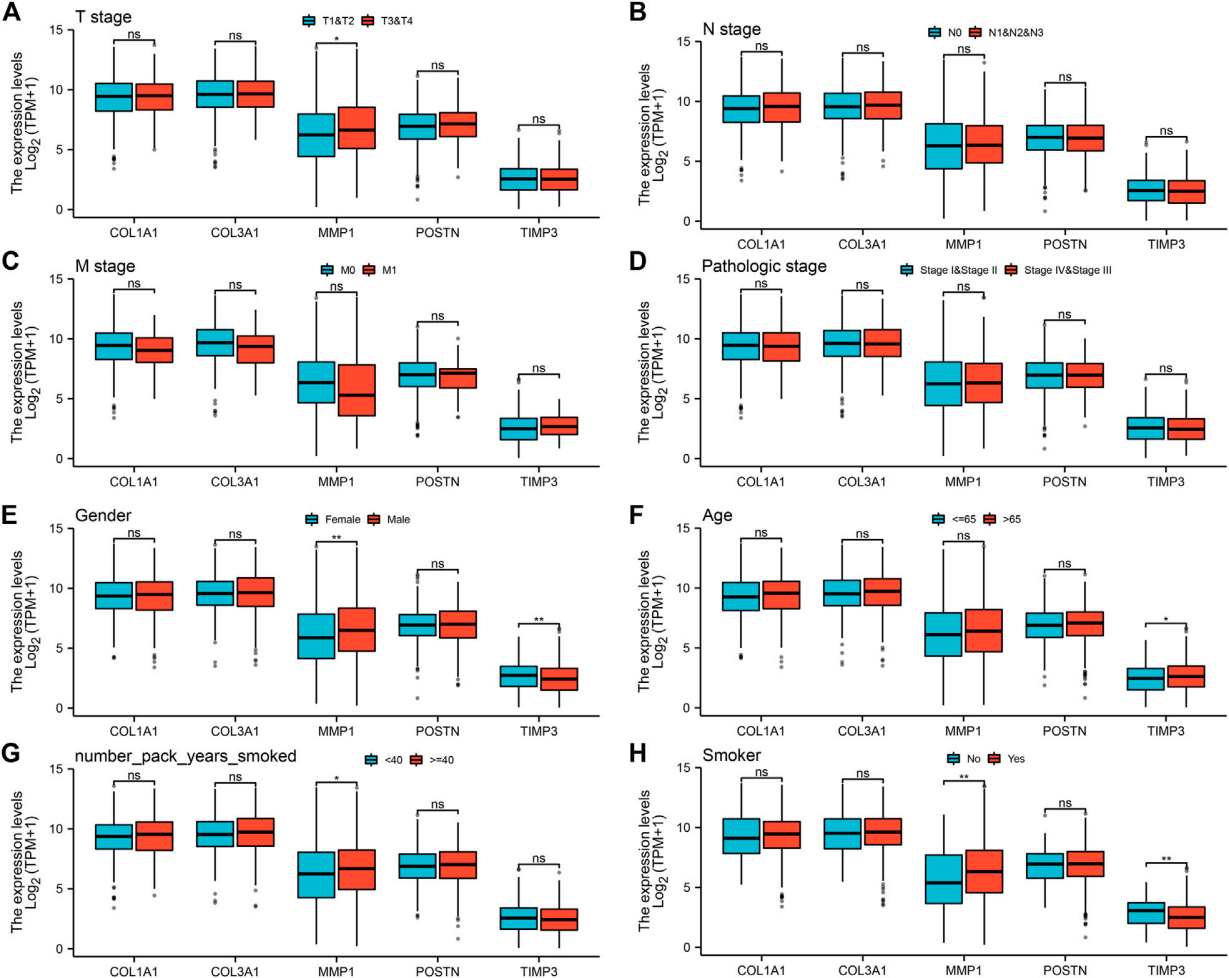
Analysis of the association between the five hub genes and clinicopathological features of NSCLC patients. Notes: **(A)**: The expression level of five hub genes in NSCLC patients with different T stage (T1-2 vs T3-4; T1-2: 872 individuals, T3-4: 162 individuals), ns = *p* > 0.05, * = *p* < 0.05, ** = *p* < 0.01, *** = *p* < 0.001; **(B)**: The expression level of five hub genes in NSCLC patients with different N stage (N0 vs N1-3; N0: 668 individuals, N1-3: 347 individuals), ns = *p* > 0.05, * = *p* < 0.05, ** = *p* < 0.01, *** = *p* < 0.001; **(C)**: The expression level of five hub genes in NSCLC patients with different M stage (M0 vs M1; M0: 773 individuals, M1: 32 individuals), ns = *p* > 0.05, * = *p* < 0.05, ** = *p* < 0.01, *** = *p* < 0.001; **(D)**: The expression level of five hub genes in NSCLC patients with different clinical stage (Stage I-II vs Stage III-IV; Stage I-II: 824 individuals, Stage III-IV: 201 individuals), ns = *p* > 0.05, * = *p* < 0.05, ** = *p* < 0.01, *** = *p* < 0.001; **(E)**: The expression level of five hub genes in NSCLC patients with different gender (Female vs Male; Female: 417 individuals, Male: 620 individuals), ns = *p* > 0.05, * = *p* < 0.05, ** = *p* < 0.01, *** = *p* < 0.001; **(F)**: The expression level of five hub genes in NSCLC patients with different age (≤ 65 vs >65; ≤ 65: 446 individuals, >65: 563 individuals), ns = *p* > 0.05, * = *p* < 0.05, ** = *p* < 0.01, *** = *p* < 0.001; **(G)**: The expression level of five hub genes in NSCLC patients with smoking years (< 40 vs ≥40; < 40: 332 individuals, ≥40: 472 individuals), ns = *p* > 0.05, * = *p* < 0.05, ** = *p* < 0.01, *** = *p* < 0.001; **(H)**: The expression level of five hub genes in smokers and non-smokers (No vs Yes; No: 93 individuals, Yes: 918 individuals), ns = *p* > 0.05, * = *p* < 0.05, ** = *p* < 0.01, *** = *p* < 0.001.

To explore the prognostic value of the five hub genes in NSCLC patients, Kaplan–Meier curves were generated. Survival analysis showed that *COL1A1, COL3A1, MMP1* and *POSTN1* were independent prognostic factors for NSCLC (Figures 4A–D). However, there was no significant difference in survival between patients with high and low TIMP3 expression (*p* > 0.05). Further analysis revealed that high MMP1 expression was correlated with T3-4 stage, male gender, and heavy smokers (*p* < 0.05, Figures 5A–H), while high TIMP3 expression was correlated with female gender, non-smokers and patients >65 of age (Figures 5E,F and Figure 5H).

### Analysis of Five Hub Genes Expression Landscape

**FIGURE 6:**
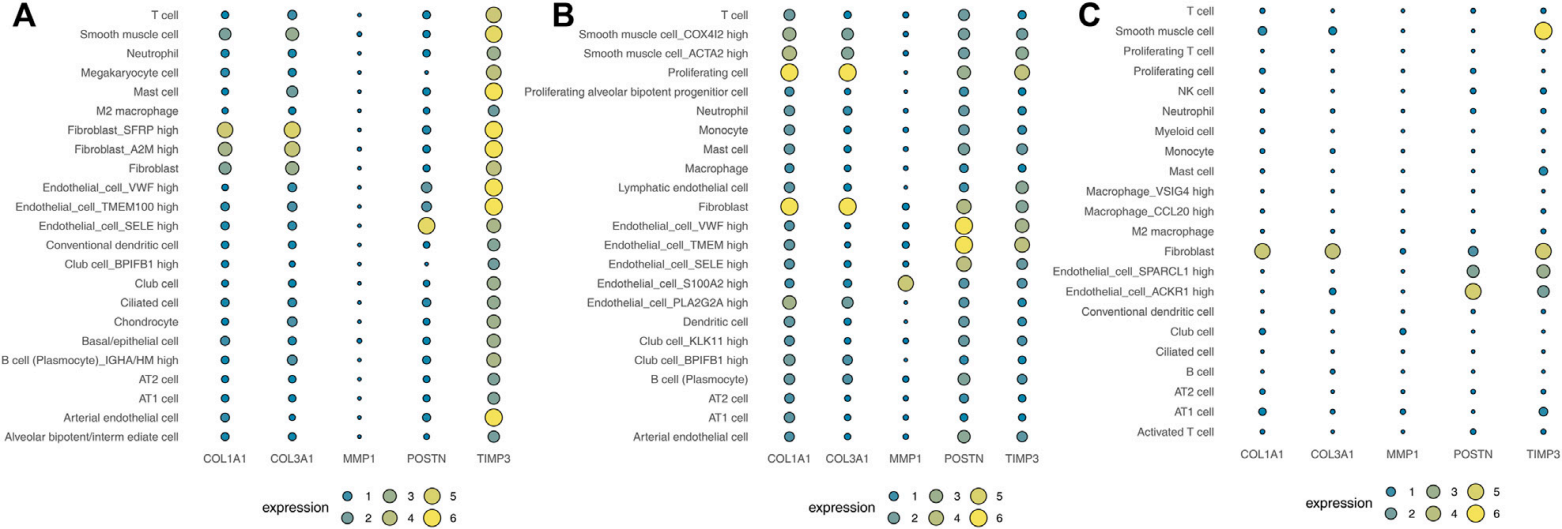
Analysis of the expression landscape of the five hub genes using Human cell landscape analysis. Notes: **(A)**: Single-cell analysis evaluating the expression of hub genes in Kidney1 based on the Human cell landscape data; **(B)**: Single-cell analysis evaluating the expression of hub genes in Kidney2 based on the Human cell landscape data; **(C)**: Single-cell analysis evaluating the expression of hub genes in Kidney3 based on the Human cell landscape data.

Human cell landscape analysis was performed to determine the expression characteristics of the five hub genes in the lung tissue of three healthy individuals (Figures 6A–C). The results indicated that *COL1A1, COL3A1, POSTN1* and *TIMP3* genes were mostly expressed in fibroblasts and smooth muscle cells. Also, we evaluated the correlation between *COL1A1, COL3A1, POSTN1, TIMP3* and the cancer associated fibroblasts based on the lung caner bulk data, showing a strong positive correlation in TCGA-LUAD and TCGA-LUSC cohorts (Figure S2, LUAD, *COL1A1*: r = 0.763, *COL3A1*: r = 0.825, *POSTN*: r = 0.743, *TIMP3*: r = 0.639; LUSC, *COL1A1*: r = 0.850, *COL3A1*: r = 0.906, *POSTN*: r = 0.878, *TIMP3*: r = 0.719). Lungmap (https://lungmap.net) provides the single-cell data of lung tissue obtained from neonate, child, and adult individuals. The result showed that *COL1A1, COL3A1, POSTN1* and *TIMP3* were mainly expressed in fibroblasts and smooth muscle cells (Figure S3). Meanwhile, the neonate lung tissue might have the highest *COL1A1* and *COL3A1* expression in fibroblasts, but the *TIMP3* was contrary. In IPF single-cell level (Figure S4, http://www.ipfcellatlas.com/), C*OL1A1, COL3A1* and *POSTN* were higher in the fibroblasts of IPF tissue compared with the normal tissue (Figure S4). In cancer single-cell level (Figure S5, http://tisch.comp-genomics.org/home/), *COL1A1, COL3A1, POSTN1, MMP3, TIMP3* were all expressed in cancer-associated fibroblasts. Meanwhile, the *TIMP3* also has a high expression level in endothelial cells (Figure S5).

### Survival Analysis Based on Cancer Associated Fibroblasts

**FIGURE 7:**
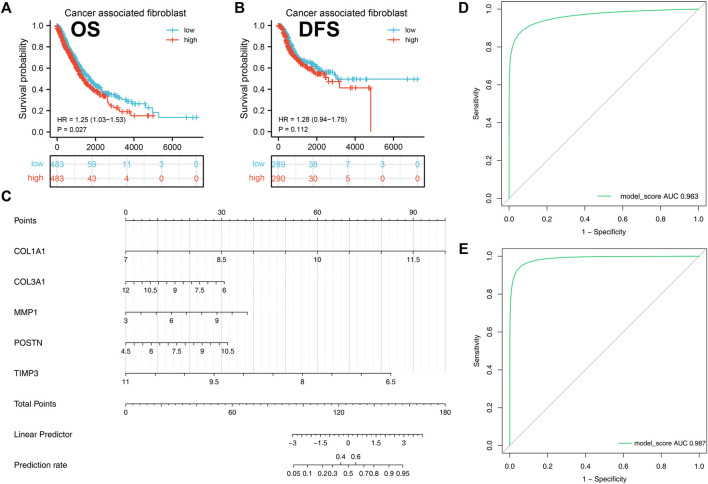
A diagnostic nomogram model was created based on the hub genes and verified in IPF and NSCLC patients. Notes: **(A–B)**: Correlation between cancer-associated fibroblasts and patients prognosis (OS and DSS); **(C)**: A diagnostic nomogram model was constructed based on the hub genes; **(D)**: The AUC curve of the model in predicting the diagnostic value of IPF; **(E)**: The AUC curve of the model in predicting the diagnostic value of NSCLC.

Kaplan-Meier analysis was then performed to investigate the prognostic value of cancer associated fibroblasts using GEPIA tool. We found that high expression of cancer associated fibroblasts was correlated with poor overall survival in NSCLC patients (*p* < 0.05, Figure 7A). Although we did not obtain the same result in disease free survival of NSCLC patients (*p* > 0.05), the data demonstrated that patients with high expression of cancer associated fibroblasts were likely to obtain unfavorable outcomes (Figure 7B).

### Establishment and Validation of Diagnostic Nomogram for IPF and NSCLC

A diagnostic nomogram was successfully constructed on the basis of the five genes and the model score was calculated as follows: *COL1A1* * 1.9135016 + *COL3A1* * −0.4915935 + *MMP1* * 0.4551232 + *POSTN* * 0.5086482 + *TIMP3* * −1.7640050 (Figure 7C). The GSE32539 dataset was used as an IPF validation group to determine the credibility of this model. The AUC value of the model score in predicting the diagnostic value of IPF was 0.963 (Figure 7D). Moreover, this model also showed a good diagnostic value for NSCLC cohorts (Figure 7E).

### Response to Immunotherapy and Chemotherapy

**FIGURE 8:**
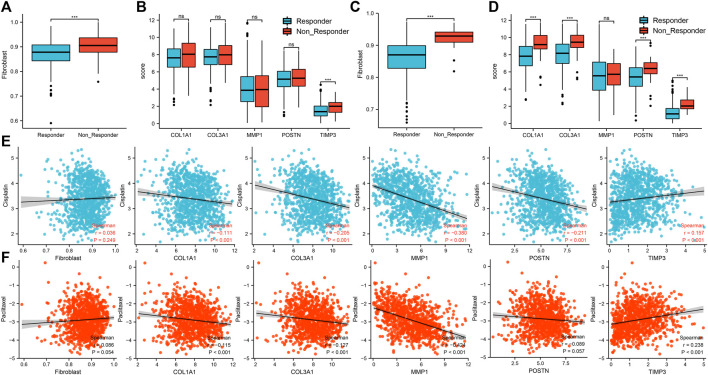
Association between the five hub genes and response to immunotherapy and chemotherapy. Notes: **(A)**: The difference of cancer-associated fibroblasts infiltration level between immunotherapy responder and non-responder patients based on the TCGA-LUAD cohort, ns = *p* > 0.05, * = *p* < 0.05, ** = *p* < 0.01, *** = *p* < 0.001; **(B)**: The difference of hub gene expression between immunotherapy responder and non-responder patients based on the TCGA-LUAD cohort, ns = *p* > 0.05, * = *p* < 0.05, ** = *p* < 0.01, *** = *p* < 0.001; **(C)**: The difference of cancer-associated fibroblasts infiltration level between immunotherapy responder and non-responder patients based on the TCGA-LUSC cohort, ns = *p* > 0.05, * = *p* < 0.05, ** = *p* < 0.01, *** = *p* < 0.001; **(D)**: The difference of hub gene expression between immunotherapy responder and non-responder patients based on the TCGA-LUSC cohort, ns = *p* > 0.05, * = *p* < 0.05, ** = *p* < 0.01, *** = *p* < 0.001; **(E)**: Correlations between cancer associated fibroblasts, five hub genes and response to cisplatin in NSCLC patients; **(F)**: Correlations between cancer associated fibroblasts, five hub genes and response to paclitaxel in NSCLC patients.

Based on the TCGA-LUAD cohort, patients with high percentage of cancer associated fibroblasts and *TIMP3* expressions were associated with resistance to immunotherapy (Figures 8A,B). Based on the TCGA-LUSC cohort, a high percentage of cancer associated fibroblasts and *COL1A1, COL3A1, POSTN1* and *TIMP3* expression was also associated with resistance to immunotherapy (Figures 8C,D). On the other hand, patients with high expression of *TIMP3* and low expression of *COL1A1, COL3A1, MMP1* and *POSTN* were associated with sensitivity to cisplatin (Figure 8E). Patients with high percentage of cancer associated fibroblasts and expression of *TIMP3* but low expression of *COL1A1, COL3A1, MMP1* and *POSTN* were associated with sensitivity to paclitaxel (Figure 8F).

## Discussion

IPF is the major type of interstitial lung disease that is characterized by progressive respiratory dysfunction which ultimately lead to death. IPF diagnosis is associated with significant increase in the incidence of NSCLC. Thus, IPF patients with coexisting NSCLC are common and there is need to increase awareness about the comorbidities.

The co-existence of IPF and lung cancer is associated with reduced overall survival, poor quality of life and lack of response to therapy (JafariNezhad and YektaKooshali, 2018). Analysis of the relationship between IPF and lung cancer reveals that similar molecular changes may be involved in inducing lung fibrosis and carcinogenesis in IPF and lung cancer, respectively (Saito et al., 2018a).

Lung tumor lesions are usually detected at the boundary of lung fibrosis, an indication that fibroblasts promote lung fibrosis and carcinogenesis (JafariNezhad and YektaKooshali, 2018) and are probably associated with the initiation of IPF and lung cancer. Squamous carcinoma is more common that adenocarcinoma in IPF patients (Ichihara et al., 2020). Several growth factors and immune cells, such as TGF-β, VEGF, and Treg cells, are involved in the progression of lung cancer and IPF (Saito et al., 2018a; Tzouvelekis et al., 2019; Shenderov et al., 2021). However, there are no effective therapeutic strategies for the treatment of IPF patients with coexisting lung cancer. The aim of this study was to identify new therapeutic targets for patients with IPF and coexisting lung cancer by using bioinformatics tools to explore common hub genes in pulmonary fibrosis and lung cancer progression.

Yu et al. explored six hub genes (*COL3A1, COL1A2, OGN, COL15A1, ASPN, MXRA5*) and sevenhub miRNAs(*hsa-let-7b-5p, hsa-miR-26a5p, hsa-miR-25-3p, hsa-miR-29c-3p, hsa-let-7c-5p, hsa-miR-29b3p, and hsa-miR-26b-5p*) that were closely associated with IPF and NSCLC (Yu et al., 2020). In his analysis, these hub genes and miRNAs were all consideredto play a role in the development of IPF and NSCLC through several pathways including extracellular matrix pathway and focal adhesion process Meantime, fibroblasts could activate the extracellular matrix pathway as well as focal adhesion process which has been thought to be related in the progression of IPF (Boutanquoi et al., 2020). In our study, we explored the DEGs between IPF and normal tissues using GSE24206, GSE53845, GSE101286 and GSE110147 IPF datasets. *COL1A1, COL3A1, MMP1, POSTN* and *TIMP3* were identified as hub genes driving the progression of IPF using STRING tool and visualized using cytoscape software analysis. The relative expression of *COL1A1, COL3A1, MMP1,* and *POSTN* was higher while *TIMP3* was lower in IPF tissue compared with normal tissue. The five genes showed a similar expression trend in TCGA-LUAD and TCGA-LUSC samples. High expression levels of *COL1A1, COL3A1, MMP1,* and *POSTN* were identified as independent prognostic risk factors associated with poor prognosis according to the median expression level. Therefore, our study demonstrated that the hub genes of IPF were independent risk factors for the progression of NSCLC.

*COL1A1* and *COL3A1* are extracellular matrix associated genes that play significant roles in the pathogenesis of lung, liver and skin fibrosis (Al-Habeeb et al., 2021; Li et al., 2021). The two genes are found extensively in connective tissues and are closely associated with Epithelial-Mesenchymal Transition (Fang et al., 2018). Sakai et al. showed TGF-b1 signaling-a prominent epithelial mesenchymal transition associated factor-induced the motility of NSCLC A549 cells and that *COL1A1* and *COL3A1* were overexpressed in parallel with active TGF β signaling (Sakai et al., 2015). In addition, *COL1A1* promotes NSCLC progression and its expression is significantly increased in IPF patients compared with NSCLC patients (Bibaki et al., 2018).

*MMP1* is a classic gene that can act as an oncogene and accelerate the progression of most cancers including NSCLC (Sauter et al., 2008). In normal processes, MMP1 breaks down the extracellular matrix and promotes the development of pulmonary fibrosis in IPF patients (Craig et al., 2015). Noteworthy, our study showed that a longer smoking history was associated with elevated *MMP1* expression levels. Tobacco is a major risk factor for IPF and progression of lung cancer (Leng et al., 2020). A recent study showed that *MMP1* expression can be induced by tobacco exposure and plays a major role in lung parenchyma destruction and pulmonary fibrosis (Adam et al., 2021). This study underlined the need for the smoking cessation for both IPF and lung cancer patients. The study of Leng et al. also demonstrated that *MMP1* might be the most potent driver gene correlated with IPF development and lung cancer progression (31).

*POSTN* is an established diagnostic biomarker for patients with IPF (Okamoto et al., 2019) that is matrix specific and is located in cancer-associated fibroblasts. *POSTIN* has prognostic value, with elevated expression levels being associated with the progression of NSCLC (Malanchi et al., 2011; Ratajczak-Wielgomas et al., 2020). Previous reports show that *TIMP3* can act as a tumor suppressor and inhibits the progression and invasion of NSCLC cells (Zhu et al., 2020). *TIMP3* is specific inhibitor for *MMP1*, and an anti-fibrotic factor that is down regulated in IPF tissues (Åhrman et al., 2018). Hence, the five genes were identified as potential hub genes associated with the transition from normal tissue to IPF tissue, and involved in NSCLC progression.

In our study, Human cell landscape analysis was performed to establish the expression characteristics of the five hub genes in healthy lung tissue at the single cell level. We found that the genes were mostly enriched in different kinds of fibroblasts and smooth muscle cells. IPF is caused by progressive and uncontrolled growth of fibroblasts, with the amount of fibroblastic foci being associated with the outcome of IPF patients (Enomoto et al., 2006). In addition, fibroblasts can promote the migration and invasion of A549 and H1299 NSCLC cell lines through the Warburg effect in coexisting culture (Koukourakis et al., 2017). Therefore, fibroblast activation is an indispensable factor contributing to the development of IPF and progression of NSCLC. According to current analysis, we purposed that these hub genes exerted their functions through fibroblasts and smooth muscle cells. However, the progression of IPF and lung cancer is a multi-factor process, there maybe several other factors playing roles in epithelial cells. For example, our analysis was based on the transcriptal data and we could not analyse the genetic mutational in epithelial cells.

Survival analysis between NSCLC patients with high and low levels of cancer-associated fibroblasts using Kaplan-Meier curves revealed that elevated levels of cancer-associated fibroblasts were correlated with unfavorable overall survival and disease free survival. These findings suggest that anti-fibrosis treatment has potential to slow down tumor progression and invasion.

We also successfully constructed a diagnostic model based on the five hub genes and obtained a high diagnostic value with the IPF validation group and NSCLC patients. IPF is diagnosed based on the exclusive diagnosis of other interstitial lung diseases including connective disease-related, drug induced and hypersensitivity pneumonitis (Guiot et al., 2017). An early diagnosis of IPF is important and this nomogram is verified to be as a potential diagnostic tool for IPF patients. Why is this signature universally applicable for both IPF and NSCLC? We speculated that the following reasons might explain this phenomena. First, Ozawa reported that after long time observation, about half of IPF patients are associated with the development of lung cancer (Ozawa et al., 2009). A majority of IPF patients died because of acute exacerbations and did not live long. IPF and lung cancer may share common pathogenic similarities including smoking, gene mutations and gene sensitivity (Ballester et al., 2019; Lee et al., 2021). Therefore, we postulated that IPF may act as a potential precancerous lesion of lung cancer and has great possibility of developing to lung cancer if follow up time is long enough. Second, fibroblasts have a major role in the development of IPF and lung carcinogenesis. These five genes are all fibrogenesis remodeling-associated genes and it is convincing a diagnostic model was successfully established. Thus, these five hub genes are all candidate target genes that promote fibroblast accumulation to induce IPF and carcinogenesis.

In our study, we also assessed the possible association between the five hub genes and response to immunotherapy and chemotherapy. Interestingly, the levels of fibroblast were closely associated with the outcome of immunotherapy. Cancer associated fibroblasts account for the majority of the solid tumor microenvironment while cancer cells only occupy a small proportion (Kratochwil et al., 2019) and are capable of secreting cytokines, such as IL-6, which suppress the activity of CD8T cells (Kato et al., 2018). Cancer associated fibroblasts are reported to be related with poor outcome of immunotherapy and poor prognosis (Barrett and Puré, 2020). Our findings were consistent with this report. Paclitaxel and cisplatin have been widely used as first line chemotherapy for NSCLC patients. Bartling et al., in 2008 reported that increased levels of fibroblasts increased invasion of H358 cells and weakened paclitaxel-induced cell death (Bartling et al., 2008). Shan et al. demonstrated that exosomal *miR-423-5p* derived from cancer-associated fibroblasts can exert its chemotherapy resistance in prostate cancer (Shan et al., 2020). Our analysis indicated fibroblasts had a positively correlation with paclitaxel and cisplatin (Spearman Correlation Coefficient was 0.036 and 0.086). We postulated that response to chemotherapy was enhanced by activation of tumor cells through the regulation of fibroblasts. In addition, the expression level of *COL1A1, COL3A1, POSTN1* and *TIMP3* were significantly associated with the response to immunotherapy in NSCLC patents, particularly LUSC patients. This maybe due to the fact that squamous cell carcinoma type most commonly occurs in IPF patients with lung cancer. GDSC demonstrated that the IC50 of cisplatin and paclitaxel were different between groups with high and low expression of the five hub genes. Among these five hub genes, *MMP1* demonstrated the highest degree of correlation with efficacy of chemotherapy (correlation coefficient was −0.380 and −0.424). *MMP1* has been already reported to cause resistance of lung adenocarcinoma to EGFR-TKIs through mTOR pathways (Saito et al., 2018b). This is the first study to demonstrate that higher MMP-1 might contribute to the resistance to chemotherapy. We could observe a weak positive correlation between fibroblast and the IC50 of cisplatin and paclitaxel. Also, *COL1A1, COL3A1* and *POSTN* showed a weak negative correlation with the IC50 of cisplatin and paclitaxel. Nevertheless, a strong positive correlation was found between the *TIMP3* expression and the IC50 of cisplatin and paclitaxel. This combined effect of *COL1A1, COL3A1, POSTN* and *TIMP3* might be the reason leading to this phenomenon.

GO analysis showed that the DEGs were mostly enriched in collagen-containing extracellular matrix, extracellular matrix organization and extracellular structure organization. These biological pathways are involved in fibrotic degradation, regeneration and remodelling. KEGG analysis demonstrated that the hub genes were enriched in the relaxin signaling pathway and amoebiasis. Relaxin pathway has been shown to mediate pulmonary fibrosis and cancer progression, and new therapeutic agents targeting this pathway are emerging (Ng et al., 2019; Chen et al., 2020). Amoebiasis could lead to parasitic pneumonia, which might be associated with pulmonary fibrosis (Cheepsattayakorn and Cheepsattayakorn, 2014). However, no direct reports reveal the underlying association between amoebiasis and pulmonary fibrosis. Therefore, it would be interesting to explore the interaction mentioned above in the future. These findings underline the importance of the relaxin pathway in IPF patients with coexisting lung cancer.

There were several limitations. First, we were unable to validate our findings using tissues resected from patients with lung cancer and IPF co-morbidities. This was because it was difficult to obtain tissues from patients as few IPF patients with lung cancer undergoing surgical resection. Most thoracic surgeons are concerned about possible acute exacerbation due to surgery. In addition, low pulmonary function hinders the surgery of IPF patients with lung cancer. Secondly, the five hub genes did not show significant association with clinicopathological parameters, a finding that needs to be validated in a large cohort. We postulated that some information can not be acquired from the TCGA datasets such as tumor grade, which may be correlated with these hub genes. Thirdly, immune dysregulation has been shown to influence the progression of IPF (Heukels et al., 2019). However, we did not consider the immune factor in this study. Fourthly, The Human Cell Landscape only provides single-cell data from the healthy lung. Therefore, the in-depth understanding of hub genes identified in lung cancer single-cell level is still unclear. Moreover, in a real biological body, the expression of *TIMP3* could be affected by thousands of regulator and cell interactions. It has a certain bias to explain the expression pattern of *TIMP3* in lung cancer only from the perspective of fibroblasts.

## Conclusion

We explored five hub genes as potential biomarkers of IPF and NSCLC progression. A diagnostic nomogram was successfully established based on the hub genes and this nomogram was verified to have high diagnostic value in IPF and NSCLC patients. The genes were also associated with the outcome of NSCLC patients and possible response to chemotherapy and immunotherapy. These genes were mostly enriched in fibroblasts and fibroblasts played a significant role in IPF and progression of NSCLC. Common signaling pathways were also explored in IPF and NSCLC. We hope that our findings can lead to the development of new therapeutic agents for IPF patients with coexisting lung cancer.

## Data Availability

The datasets presented in this study can be found in online repositories. The names of the repository/repositories and accession number(s) can be found in the article/Supplementary Material.

## Supplementary Material

Supplementary Figure S1 Background correction and data standardization of four IPF raw gene expression datasets. Notes: **(A)**: Background correction and data standardization of GSE24206; **(B)**: Background correction and data standardization of GSE53845; **(C)**: Background correction and data standardization of GSE101286; **(D)**: Background correction and data standardization of GSE110147.

Supplementary Figure S2 The correlation between cancer-associated fibroblast and *COL1A1, COL3A1, POSTN, TIMP3.* Notes: **(A-D)**: The correlation between cancer-associated fibroblast and *COL1A1, COL3A1, POSTN, TIMP3* in TCGA-LUAD cohort; E-H: The correlation between cancer-associated fibroblast and *COL1A1, COL3A1, POSTN, TIMP3* in TCGA-LUSC cohort.

Supplementary Figure S3 The expression pattern of COL1A1, COL3A1, POSTN, TIMP3, and MMP1 in normal lung tissue.

Supplementary Figure S4 The expression pattern of *COL1A1, COL3A1, POSTN, TIMP3,* and *MMP1* in IPF tissue.

Supplementary Figure S5 The expression pattern of *COL1A1, COL3A1, POSTN, TIMP3*, and *MMP1* in lung cancer tissue.
