# Supplementary material for: Identification of Hub Genes in Idiopathic Pulmonary Fibrosis and NSCLC Progression:Evidence From Bioinformatics Analysis
Source: Front Genet. 2022 Apr 11;13:855789. doi: 10.3389/fgene.2022.855789 (PMC9038140; doi:10.3389/fgene.2022.855789)
Supplement: Supplementary file 1 [file Table1.docx]

| **Table S1. The 31 DEGs whose expression overlapped among the four datasets** |
| --- |
| *IL1R2, FAM107A, SLCO4A1, NECAB1, LRRC17, LRRN1, COL3A1, CFH, S100A12, BCHE, PEBP4, CDH3, BTNL9, COL1A1, MMP7, CXCL14, SFRP2, IL13RA2, SULF1, CDH13, CA4, HSD17B6, TIMP3, THBS2, GPR87, POSTN, MSMB, MMP1, CLDN18, FCN3, GREM1* |
