# Supplementary figures and images for: Identification of Hub Genes in Idiopathic Pulmonary Fibrosis and NSCLC Progression:Evidence From Bioinformatics Analysis
Source: Front Genet. 2022 Apr 11;13:855789. doi: 10.3389/fgene.2022.855789 (PMC9038140; doi:10.3389/fgene.2022.855789)

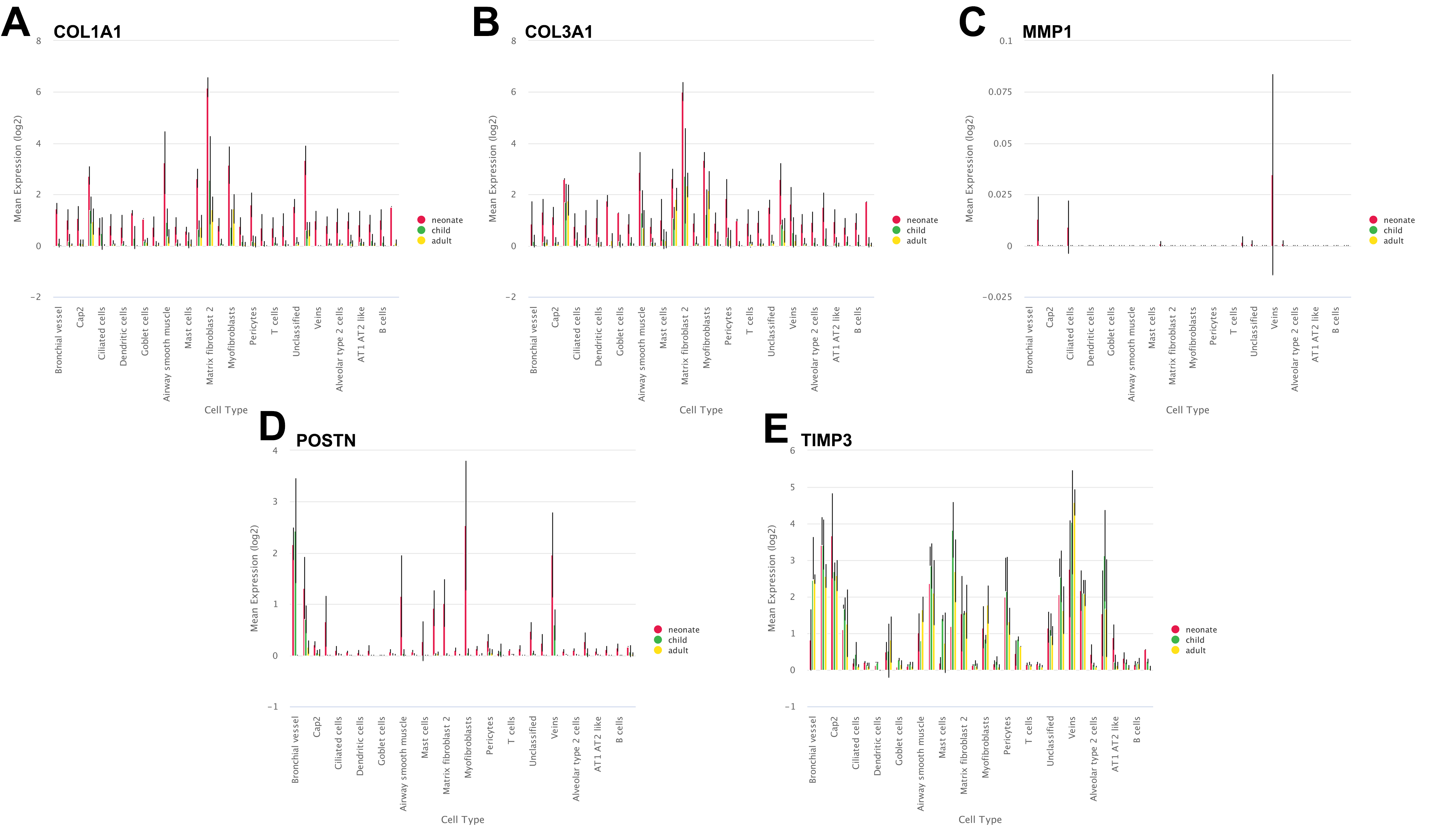

Supplement: Supplementary file 2 [file Image3.tif]

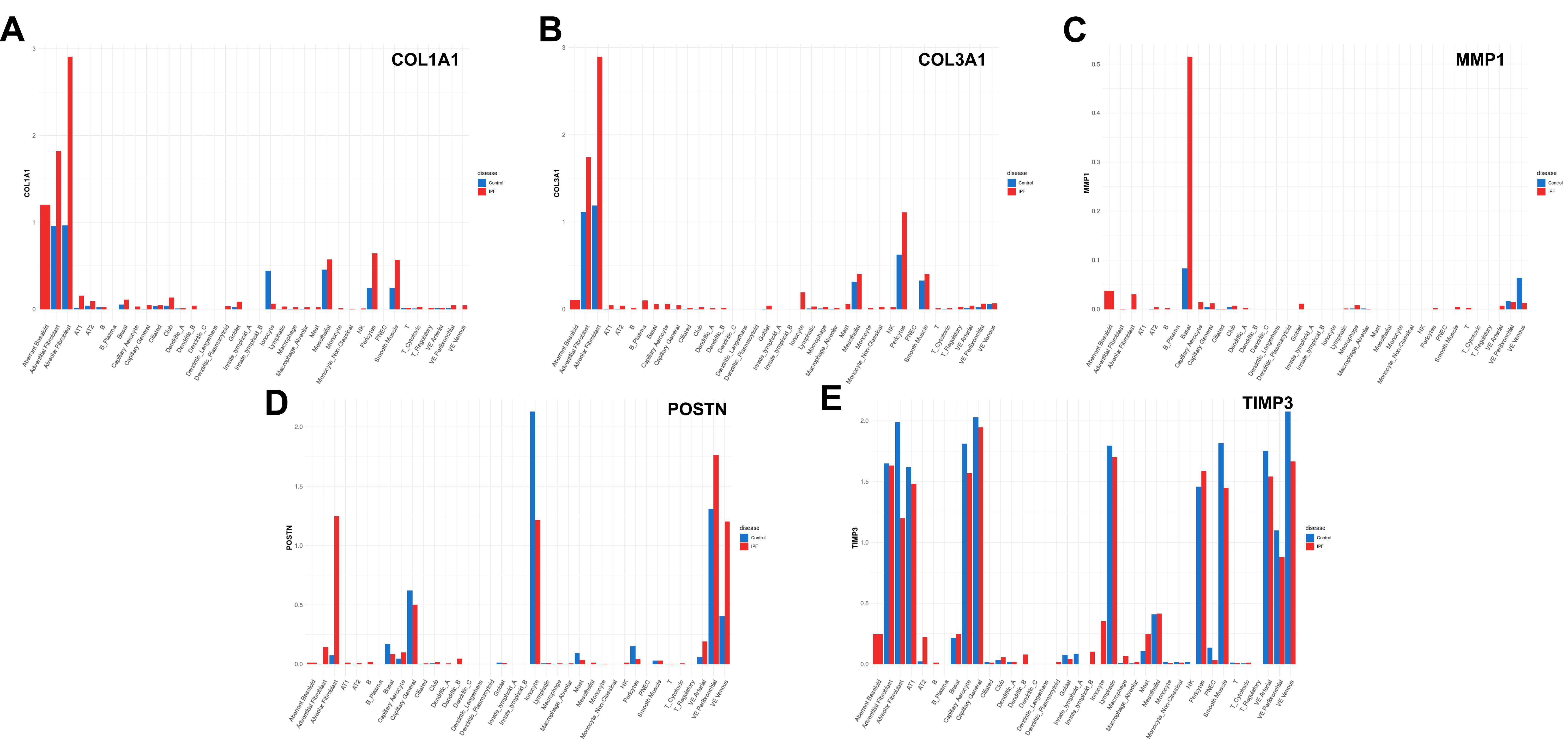

Supplement: Supplementary file 3 [file Image4.tif]

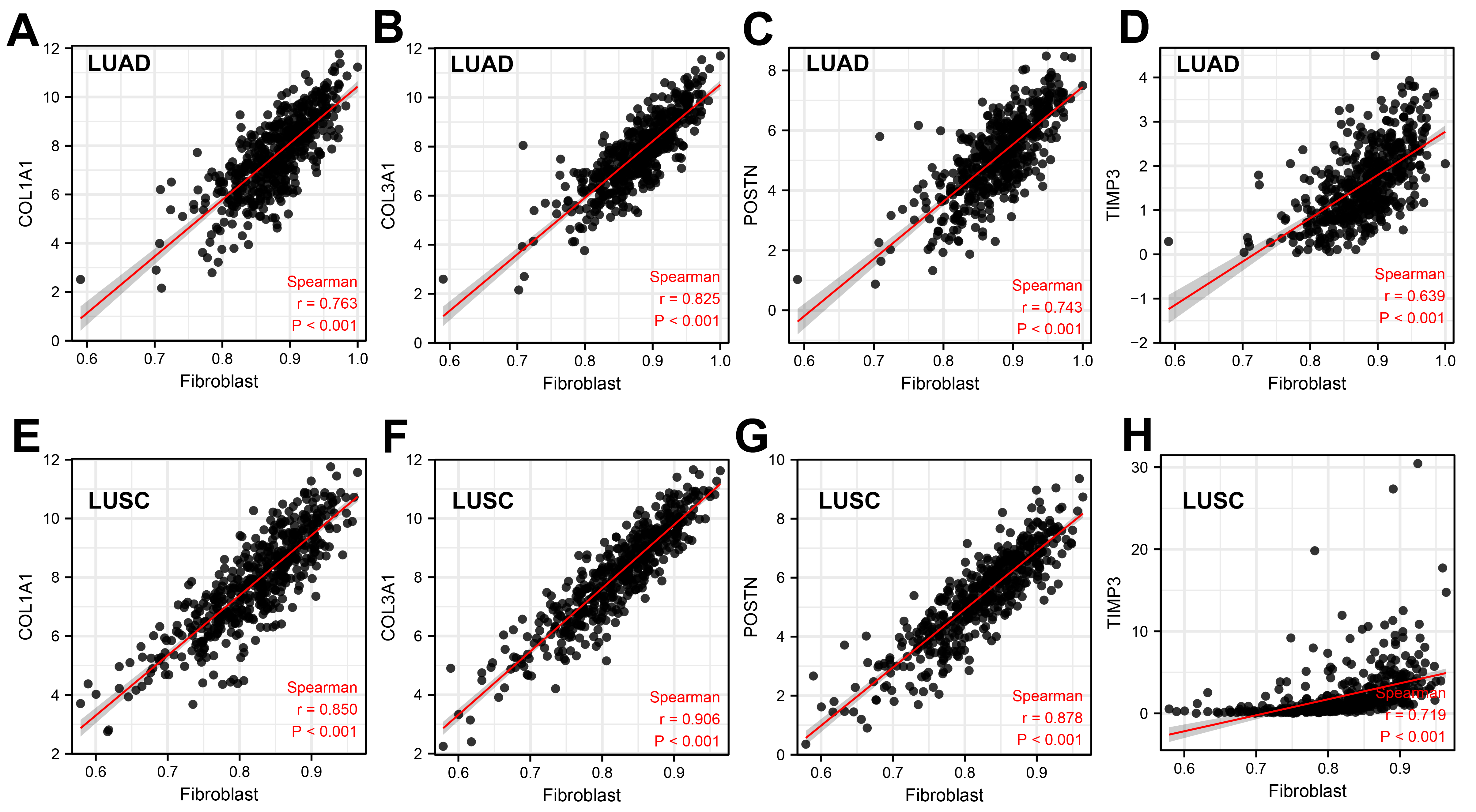

Supplement: Supplementary file 4 [file Image2.tif]

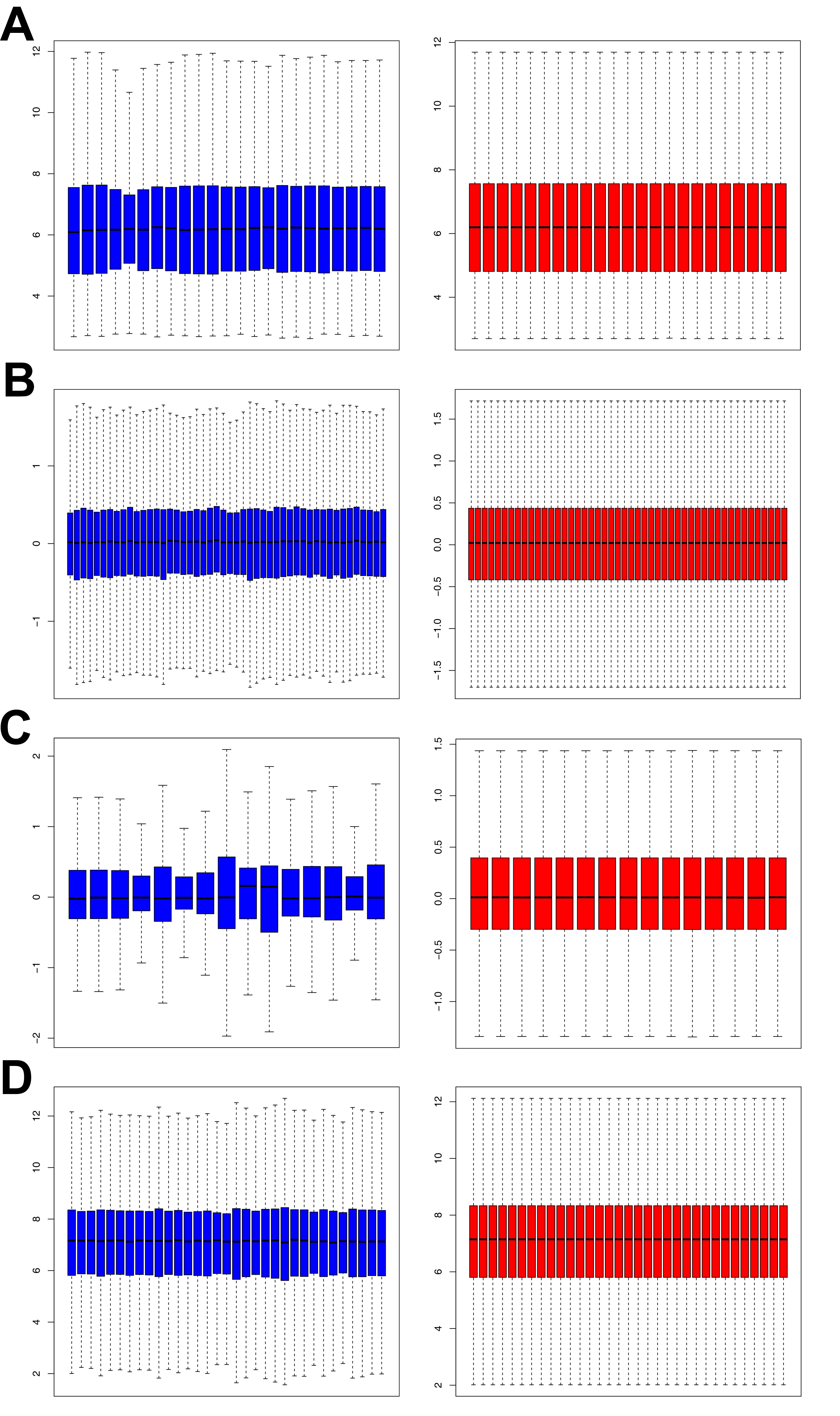

Supplement: Supplementary file 5 [file Image1.tif]

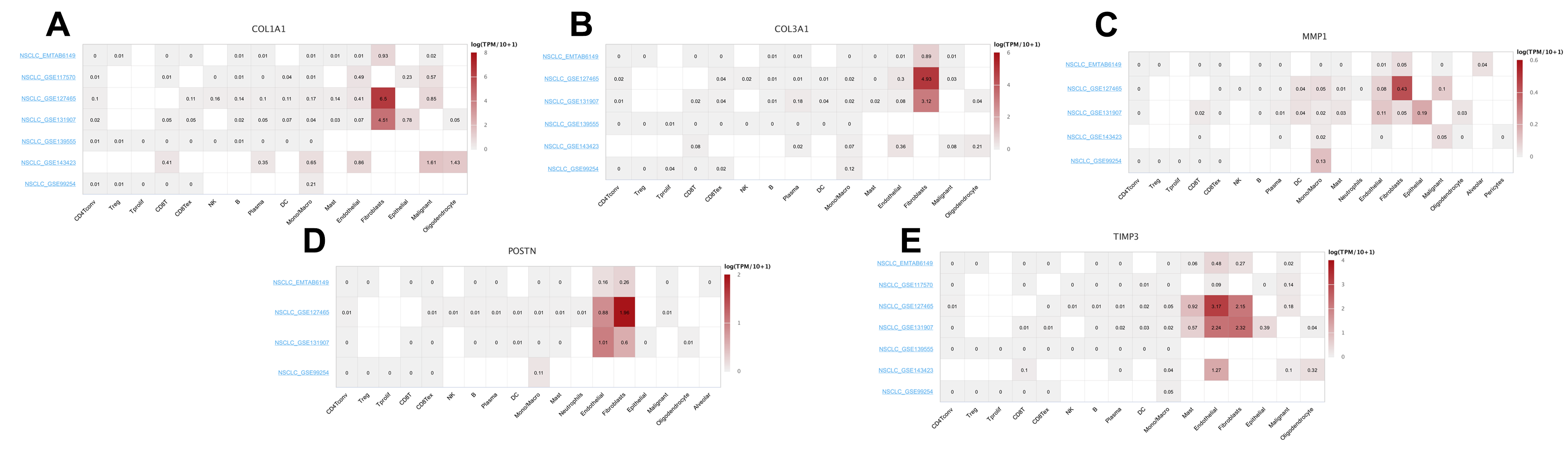

Supplement: Supplementary file 8 [file Image5.tif]
