# Supplementary material for: Identification of Hub Genes in Idiopathic Pulmonary Fibrosis and NSCLC Progression:Evidence From Bioinformatics Analysis
Source: Front Genet. 2022 Apr 11;13:855789. doi: 10.3389/fgene.2022.855789 (PMC9038140; doi:10.3389/fgene.2022.855789)
Supplement: Supplementary file 6 [file Table2.docx]

| **Table S2. The complete information of biological enrichment pathways based on 31 DEGs** | | | | | | |
| --- | --- | --- | --- | --- | --- | --- |
| ONTOLOGY | ID | Description | GeneRatio | Genes | pvalue | qvalue |
| BP | GO:0030198 | extracellular matrix organization | 8/30 | *COL1A1/COL3A1/MMP1/MMP7/SFRP2/POSTN/SULF1/GREM1* | 8.46e-08 | 5.44e-05 |
| BP | GO:0043062 | extracellular structure organization | 8/30 | *COL1A1/COL3A1/MMP1/MMP7/SFRP2/POSTN/SULF1/GREM1* | 2.41e-07 | 7.76e-05 |
| BP | GO:0060348 | bone development | 6/30 | *COL1A1/SFRP2/LRRC17/SULF1/GREM1/CLDN18* | 1.08e-06 | 2.32e-04 |
| BP | GO:0030199 | collagen fibril organization | 4/30 | *COL1A1/COL3A1/SFRP2/GREM1* | 1.62e-06 | 2.60e-04 |
| BP | GO:0060346 | bone trabecula formation | 2/30 | *COL1A1/GREM1* | 1.11e-04 | 0.011 |
| CC | GO:0062023 | collagen-containing extracellular matrix | 9/31 | *CDH13/COL1A1/COL3A1/SFRP2/THBS2/TIMP3/POSTN/SULF1/GREM1* | 8.22e-09 | 3.98e-07 |
| CC | GO:0005583 | fibrillar collagen trimer | 2/31 | *COL1A1/COL3A1* | 1.30e-04 | 0.002 |
| CC | GO:0098643 | banded collagen fibril | 2/31 | *COL1A1/COL3A1* | 1.30e-04 | 0.002 |
| CC | GO:0005581 | collagen trimer | 3/31 | *COL1A1/COL3A1/FCN3* | 3.41e-04 | 0.004 |
| CC | GO:0098644 | complex of collagen trimers | 2/31 | *COL1A1/COL3A1* | 4.02e-04 | 0.004 |
| MF | GO:0005539 | glycosaminoglycan binding | 5/29 | *CFH/MMP7/THBS2/POSTN/SULF1* | 3.20e-05 | 0.001 |
| MF | GO:0005201 | extracellular matrix structural constituent | 4/29 | *COL1A1/COL3A1/THBS2/POSTN* | 1.38e-04 | 0.002 |
| MF | GO:0048407 | platelet-derived growth factor binding | 2/29 | *COL1A1/COL3A1* | 1.41e-04 | 0.002 |
| MF | GO:0008201 | heparin binding | 4/29 | *CFH/MMP7/THBS2/POSTN* | 1.58e-04 | 0.002 |
| MF | GO:1901681 | sulfur compound binding | 4/29 | *CFH/MMP7/THBS2/POSTN* | 6.99e-04 | 0.006 |
| KEGG | hsa05146 | Amoebiasis | 3/16 | *COL1A1/COL3A1/IL1R2* | 9.72e-04 | 0.028 |
| KEGG | hsa04926 | Relaxin signaling pathway | 3/16 | *COL1A1/COL3A1/MMP1* | 0.002 | 0.028 |
